# The impact of COVID-19 vaccination on glycaemic control in children and adolescents with type 1 diabetes mellitus on continuous glucose monitoring

**DOI:** 10.1007/s00592-022-01968-y

**Published:** 2022-09-07

**Authors:** Niki Gouda, Meropi Dimitriadou, Georgia Sotiriou, Athanasios Christoforidis

**Affiliations:** 1st Paediatric Department, School of Medicine, Faculty of Health Sciences, Aristotle University of Thessaloniki, Ippokratio General Hospital, 49 Konstantinoupoleosstr, 54642 Thessaloniki, Greece

**Keywords:** Type 1 diabetes mellitus, Children, Adolescents, SARS-Cov-2, Vaccination, COVID-19

## Abstract

**Aims:**

To investigate the impact of SARS-COV-2 vaccination on the glycaemic control in children and adolescents with T1DM wearing continuous glucose monitoring (CGM).

**Methods:**

Caregivers of children and adolescents with T1DM were questioned regarding SARS-CoV-2 vaccination during their regular visits at the Pediatric Diabetes Outpatient Clinic. Data regarding Time in Range (TIR) (glucose levels: 70–180 mg/dl) 7 days prior and 7 days after a vaccination dose were collected in patients wearing CGM and data regarding insulin daily doses were also obtained for the insulin pump users.

**Results:**

From a total of 135 patients eligible for SARS-CoV-2 vaccination, 70 (51.9%) children (37 boys, 52.9%) were vaccinated with at least one dose. Seven patients received only one dose, whereas two children received a third booster shot. No statistically significant difference was observed in either TIR (64.19% post vs. 65.53% pre, *p* = 0.158) or total daily insulin dose (40.08 U/day post vs. 39.32 U/day pre, *p* = 0,282). Additionally, in ten patients on Hybrid Closed-Loop System the percentage of the automated insulin boluses given post-vaccination was not statistically significant different compared to the boluses given pre-vaccination (15.80% vs. 16.90%, *p* = 0,491).

**Conclusions:**

Vaccination against SARS-CoV-2 in children and adolescents with T1DM is safe and is not associated with immediate glucose imbalance.

## Introduction

Since March 2020, the world has been facing the COVID-19 pandemic with detrimental and fatal consequences, especially for elderly people, but also for people with specific diseases regardless of age [1]. In general, patients with Diabetes Mellitus are considered as a vulnerable group for developing severe COVID-19 [2], although in young patients with uncomplicated Type 1 Diabetes Mellitus (T1DM) no evidence suggest a higher risk for serious illness [3].

The vaccination campaign against SARS-CoV-2 launched at the dawn of 2021 with prioritization of elderly and vulnerable groups and gradually being expanded to all. In Greece, SARS-CoV-2 vaccination of children aged 5–12 years old started on 15 December 2021, while a couple of months earlier the vaccine was available for age groups 15–18 years and 12–15 years.

Vaccine hesitancy against COVID-19, mainly attributed to safety or side effects concerns but also associated to religiosity and even sociopolitical beliefs, led to poor vaccination coverage rates [4]. Additionally, low rates of severe disease and death associated with SARS-CoV-2 infection in children and young people led to even lower vaccination coverage rates in these age groups [5]. For patients with diabetes, vaccine hesitancy is also driven by unjustifiable concerns of temporary instability of blood glucose levels post-vaccination [6]. A recent report has linked SARS-CoV-2 vaccination with Diabetic Ketoacidosis (DKA) in two young Indian adults with poorly-controlled T1DM [7]. Furthermore, there were reports of vaccine—induced hyperglycaemia that led to complications and need for hospitalization in adult patients with T2DM, presented with symptoms of hyperglycaemia or even DKA and hyperglycaemic hyperosmolar syndrome [8]. However, more rigorous evidence with the means of continuous glucose monitoring investigated post-vaccination glycaemic control on adult patients with diabetes showing conflict results [9, 10].

The aim of this study was to investigate the impact of SARS-COV-2 vaccination on the glycaemic control in children and adolescents with Type 1 Diabetes Mellitus, on Continuous Glucose Monitoring (CGM) and to identify possible associations to demographic or other parameters.

## Methods

During March 2022, caregivers of children and adolescents with T1DM were approached and questioned regarding SARS-CoV-2 vaccination during their regular visits at the Pediatric Diabetes Outpatient Clinic of the first Pediatric Department at Ippokratio General Hospital of Thessaloniki. 3 months after the initiation of vaccination for the younger age group, we thought that the percentage of patients vaccinated at least with one dose would adequately reflect the percentage of parents’ willingness to vaccinate their children. However, the primary outcome of the study was determined as the comparison of the percentage of time in range (glucose levels: 70–180 mg/dl) 7 days prior and 7 days after a vaccination dose with the use of continuous glucose monitoring system. Eligibility criteria for entering a patient in the final analysis were: (i) T1DM diagnosis, (ii) age: 5–18 years, (iii) use of continuous glucose monitoring system, (iv) at least one dose of SARS-CoV-2 vaccine, 7 days prior to visit, (v) glucose data availability 7 days prior and 7 days after a vaccination dose and (vi) willingness to participate. Children and their caregivers were informed for the nature and the purpose of the study and a verbal consent was obtained for every participant. The study was performed in accordance with the Helsinki Declaration of 1975 and was approved by the Scientific and Administrative Council of Aristotle University of Thessaloniki as a postgraduate dissertation.

Demographic data including date of birth, date of T1DM diagnosis, insulin regimes were retrieved from patients’ medical files. Additionally, data on glycaemic control and insulin doses were downloaded from Medtronic Carelink platform for patients wearing Medtronic MiniMed 640G or Medtronic MiniMed 780G insulin pumps accompanied with Enlite™ Sensor and Guardian™ 2 Link or Guardian™ Sensor 3, respectively. For FreeStyle® Libre users, data on glycaemic control were obtained from the Libreview platform. For the two time-intervals selected (a week prior to vaccination and a week after) data collected included “Time in Range (TIR)” percentage (%), mean glucose levels (mg/dl), mean total daily insulin dose administered (u), percentage of insulin given as bolus (%), and for those on Hybrid Closed-Loop System (Medtronic MiniMed780G insulin pump) the percentage of automated insulin bolus given (%). For patients on Multiple Daily Injection regimes, insulin doses could not accurately collected retrospectively, thus, they were not included in the analysis.

For statistical analysis and graphical demonstration IBM corp. SPSS Statistics® version 24.0.0.0 were employed. The results are presented as means ± Standard Deviation (SD). The Kolmogorov–Smirnov test was used for assessing the normality of the studied parameters. Comparison of the means was performed with Student’s *T* test for paired samples and Wilcoxon Signed Ranks Test for parameters with normal and skewed distribution, respectively. In parameters with normal distribution linear correlations were calculated with the Pearson’s correlation coefficient, whereas Spearman’s correlation coefficient was employed for nonparametric variables. A *P* value of 0.05 or less was considered statistically significant.

## Results

**Fig. 1:**
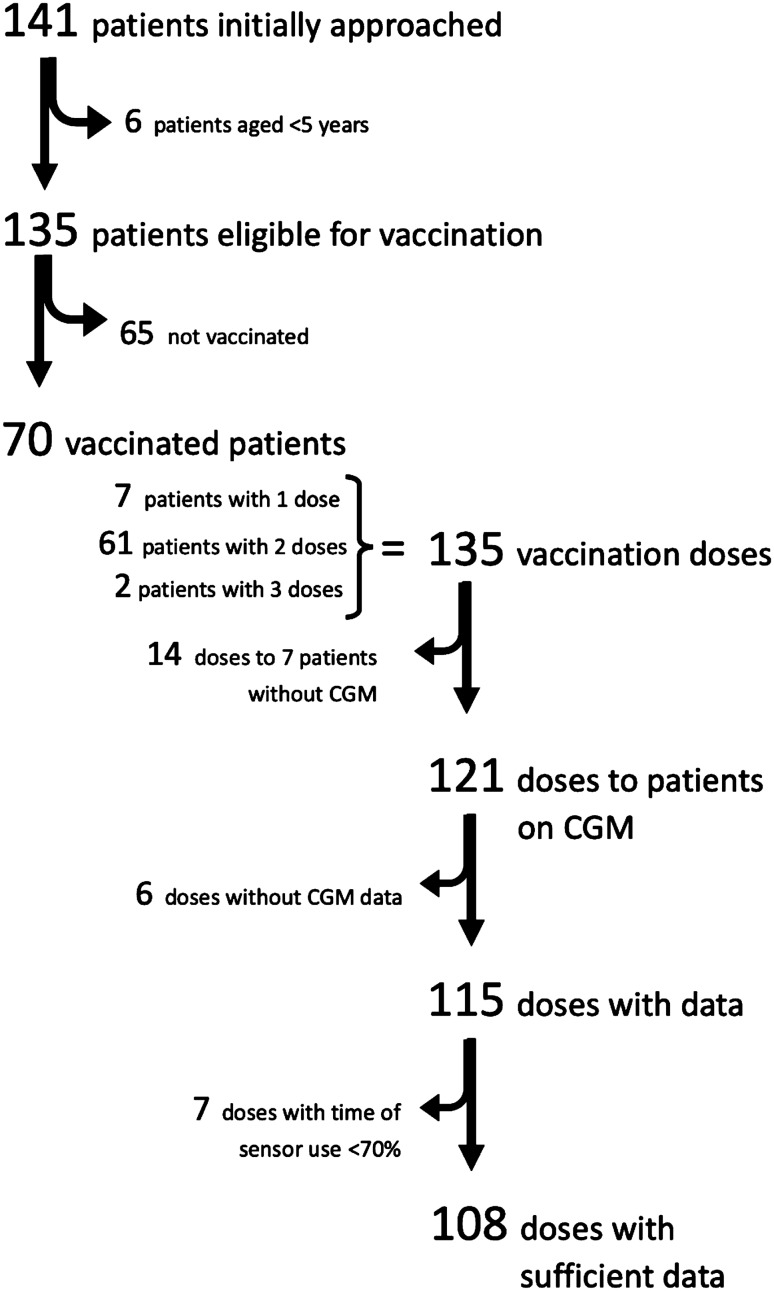
Flow chart of the study population selection including patient recruitment, exclusion criteria, leading to single doses of SARS-CoV-2 vaccination with available and sufficient data on CGM

Figure 1 shows a flow chart of the study population recruitment. A total of 141 children were initially approached, six were under the age of five and were not yet eligible for SARS-Cov-2 vaccination, thus 135 were eventually recruited (63 boys, 46.7%) with a mean age of 11.68 ± 3.50 years. Seventy (51.9%) children (37 boys, 52.9%) were vaccinated with at least one dose with an average age of 12.56 ± 3.37 years, seven of them received only one dose, 61 received two doses, whereas two children received a third booster shot. Sixty-five children (26 boys, 40.0%) were not vaccinated at all (average age 10.73 ± 3.41 years). Most of the unvaccinated children had a confirmed SARS-CoV-2 infection (*n* = 35/65, 53.84%), but for many of them the infection came long after the date SARS-CoV-2 vaccine was available for their age group. Demographic characteristics of the study population and data regarding CGM use and insulin regimes are shown in Table 1.

**Table 1:** Characteristics of the study population stratified as vaccinated and non-vaccinated

	Vaccinated	Non-vaccinated
*n* (%)	70 (51.85)	65 (48.15)
Male (*n*, %)	37 (52.86)	26 (40.00)
Age (years ± SD)	12.56 ± 3.37	10.73 ± 3.41
Age at diagnosis (years ± SD)	7.59 ± 3.63	7.05 ± 3.64
*Insulin pump (n,%)*	37 (52.86)	14 (21.54)
Medtronic Minimed G640	27	12
Medtronic Minimed G780	10	2
*CGM*	65 (92.86)	55 (84.62)
FreeStyle® Libre	31	41
Enlite	2	2
Guardian 2	22	10
Guardian 3	10	2
No CGM	5	10
*# vaccination doses*		
1 dose (*n*, %)	7 (10.00)	–
2 doses (*n*, %)	61 (87.14)	–
3 doses (*n*, %)	2 (2.86)	–

A total of 135 vaccination doses were performed (one dose in seven persons, two doses in 61 persons and three doses in two persons). Among the seven persons that did not receive the second vaccine dose, six were infected before the scheduled date of their second dose, and one had been infected earlier, and hence received only one dose, according to the current guidelines. Seven patients that received two doses of the vaccine did not wear CGM device, therefore no data were analysed. In a total of six doses, glucose monitoring data could not be obtained mainly due to time lapse or incapability for successful data upload. In seven doses, data were not analysed as there was not sufficient time of sensor use (> 70%) either in the week prior or post-vaccination or both. Thus, data on glycaemic control on a total of 108 vaccination doses were included in the final analysis. Figure 1 shows also a flow chart of data available on vaccination doses.

**Fig. 2:**
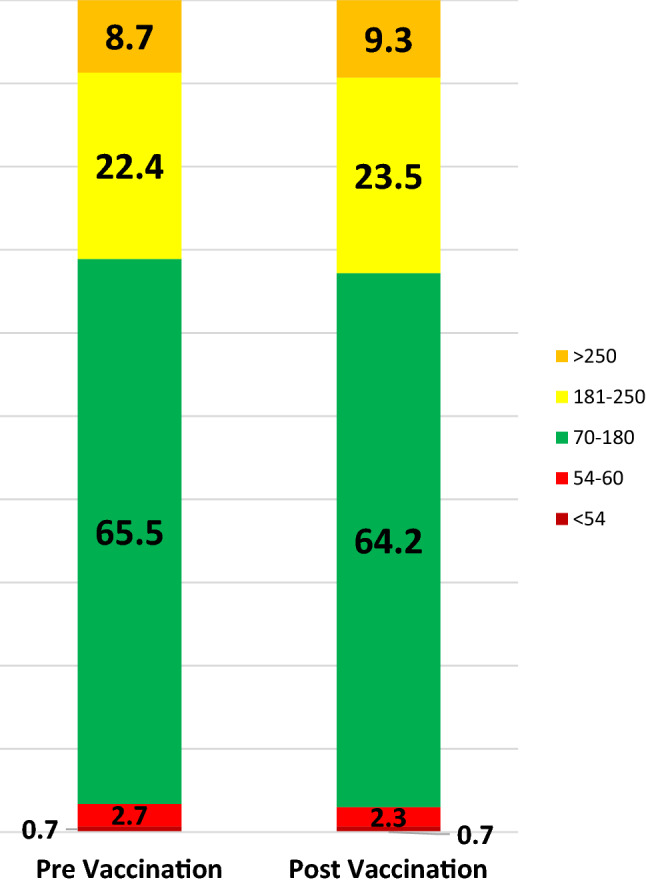
Time in range a week prior and a week after vaccination for a total of 108 vaccination doses

Data on glycaemic control one week before the vaccination and one week after are presenting in Table 2 and Fig. 2. The Time in Range (70–180 mg/dl) demonstrated a slight decrease (64.19 ± 13.81% after vaccination vs. 65.53 ± 14.35% the week before) which was not statistically significant (*p* = 0.158). Similarly, the average glucose level increased the week following the vaccination, but again the difference was not statistically significant (160.53 ± 23.68 mg/dl vs. 156.68 ± 26.96 mg/dl, *p* = 0.057). Finally, no difference in TIR change post-vaccination was associated to weather it was the first dose or a subsequent dose (Table 1).

**Table 2:** Comparative data of one week before and one week after vaccination (data are presented as mean values ± SD)

Parameter	Week before vaccination	Week after vaccination	*P* value
TIR % (*n* = 108)	65.53 ± 14.35	64.19 ± 13.81	0.158
Average glucose level (mg/dl) (*n* = 108)	156.68 ± 26.96	160.53 ± 23.68	0.057
Daily insulin (U/kg/d) (*n* = 61)	0.68 ± 0.03	0.69 ± 0.03	0.282
Bolus insulin (%) (*n* = 61)	60.03 ± 7.58	58.83 ± 10.37	0.311
Automated bolus (%) (*n* = 10)	16.90 ± 10.71	15.80 ± 12.28	0.491
TIR %—first dose (*n* = 52)	65.46 ± 14.85	64.27 ± 13,13	0.340
TIR %—second or third dose (*n* = 56)	65.59 ± 13.93	64.10 ± 14.64	0.305

In 61 vaccinations, data from insulin pump regarding the mean total daily dose of insulin given were studied comparing the week before with the week after the vaccination. Again, no statistically significant increase was recorded in the insulin administered the week after vaccination (0.69 ± 0.03 U/kg/d the week after vs. 0.68 ± 0.03 U/kg/d the week before, *p* = 0.282). Neither was there any significant increase in the percentage of insulin administered as bolus (58.83 ± 10.37% the week after vs. 60.03 ± 7.58% the week before, *p* = 0.311). Finally, in ten patients on Hybrid Closed-Loop Insulin Delivery System the percentage of the automated insulin boluses given the week following the vaccination was not statistically significant different compared to the boluses given the week before (15.80 ± 12.28% after vs. 16.90 ± 10.71% before, *p* = 0.491).

## Discussion

Our results showed that SARS-CoV 2 vaccination had no effect on glycaemia in children with T1DM as this was assessed by means of continuous glucose monitoring and for a weekly interval. A slight decrease in TIR accompanied by an increased in mean glucose levels in post-vaccination week, both statistically insignificant possibly designated by the limited study population, was observed, however with no clinical impact. To our knowledge, this is the first report of glucose profile immediate after SARS-CoV 2 vaccination on paediatric population with T1DM. Previous reports on adults with T1DM have shown conflict results. Heald et al. [10] have reported significantly decreased TIR following SARS-CoV 2 vaccination in 97 adults with T1DM, whereas Aberer et al. [9] in their study including 58 adults with T1DM, find no significant difference in TIR for 2 days prior and 3 days after SARS-CoV 2 vaccination. Of particular interest is that in the study by Heald et al., a reverse association between glycaemic control and TIR decrease post-vaccination was noted as patients with lower ΗbA1c had greater TIR reduction. However, in both studies showing no significant TIR reduction (Aberer’s and ours) glycaemic control of the studied population was superior compared to the study by Heald et al., as this was indicated by higher pre-vaccination TIR. On the other hand, a significant reverse correlation between TIR pre-vaccination and TIR reduction post-vaccination was supported by our results as well.

As our group of patients and caregivers are well trained in dealing with glucose fluctuations, could this post-vaccination euglycaemia be attributed to frequent insulin corrections? To answer this inquiry, we analysed data from insulin pump users and find that no statistically increased insulin dose was given to post-vaccination. Moreover, in ten patients on hybrid closed-loop system automated bolus insulin, as an indicator of hyperglycaemia-mediated automated insulin deliver did not differ in the post-vaccination week. This finding is original in the literature.

With regard to the type of vaccine, our population was exclusively vaccinated with Pfizer/BioNTech vaccine, using mRNA technology, as this was the only currently approved for this age range. Also, in the study by Aberer’s et al. [9] mRNA vaccines (Pfizer/BioNTech and Moderna) were used in the majority of the participants (93%) versus 7% used the AstraZeneca vaccine. In the British study by Heald et al. [10] showing significant post-vaccination glucose perturbation, AstraZeneca vaccine was given in 52 patients versus 45 vaccinated with Pfizer/BioNTech vaccine. However, no difference on post-vaccination glycaemia was shown in the sub-analysis based on the type of the vaccine. Additionally, our study showed that no difference in post-vaccination glycaemic status was associated to the vaccination dose number. This information is original as all data in the aforementioned studies referred solely to the first dose of the vaccine. Finally, in the British study by Heald et al. [10] TIR reduction post-vaccination was more prominent in patients also receiving oral medication and in their preliminary but limited report age was a predisposing factor for glucose variability after vaccination [11]. Both parameters could not be investigated in our population, as we studied paediatric population and oral medication in not used as an adjunctive therapy for T1DM in children.

As for the time period studied pre and post-vaccination, we chose the weekly interval, in-line with the methodology followed by Heald et al. [10] and this was arbitrary set based on the sporadically and anecdotally report of some patients referring to weekly glucose instability. Aberer et al. [9] limited the study interval to 2 days prior and 3 days post-vaccination and they based their decision to the duration of possible side effects, e.g. increased body temperature, headache, body ache, fatigue, and injection site reaction. They also created a score based on side effects and TIR reduction was more prominent in T1DM patients experienced more pronounced side effects. In our study, the most often self-reported side effect was ache at the vaccination site, but no correlation to TIR reduction was observed (data are not shown).

Despite the fact that compared to adults, both the incidence and the severity of COVID-19 are lower in paediatric population, recent epidemiological studies support the fact that the risk might be greater than initially thought [12]. Additionally, complications from long COVID or post COVID Multisystem Inflammatory Syndrome (MIS-C) can be detrimental [13]. Moreover, distancing and staying away from school and activities due to quarantine can affect mental health and well-being [12]. For all these reasons, vaccination against SARS-CoV 2 is highly recommended even in healthy paediatric population. With regard to its efficacy, a recent report has shown that two doses of SARS-CoV2 vaccine for T1DM patients is comparably efficacious to healthy controls [14]. However, vaccine hesitancy among patients with both type 1 and type 2 diabetes is not negligible and seems to be more prevalent in individuals with lower adherence to medical prescriptions and/or reduced concerns over their health [15]. Some individuals base their refusal for vaccination on concerns regarding acute side effects and mainly glucose disturbances. Our study results come to dispute these unjustified concerns.

In conclusion, our study showed that vaccination against SARS-CoV-2 in children and adolescents with T1DM is safe and is not associated with immediate glucose imbalance. Thus, no concerns and hesitancy against vaccination should be based on unjustified evidence regarding post-vaccination glucose variability.
